# Mesoglycan for pain control after open excisional HAEMOrrhoidectomy (MeHAEMO): an observational multicentre study on behalf of the Italian Society of Colorectal Surgery (SICCR)

**DOI:** 10.1186/s12893-020-00914-5

**Published:** 2020-10-22

**Authors:** G. Gallo, S. Di Saverio, G. Clerico, A . Sturiale, M. Manigrasso, A. Realis Luc, M. Trompetto, G. Sammarco, the MeHAEMO Working Group, Francesco Ferrari, Antonio Carpino, Giuseppe Sena, Giuseppina Vescio, Emanuela Stratta, Alberto Realis Luc, Giuseppe Clerico, Mario Trompetto, Paolo Tonello, Silvia Cornaglia, Vincenzo Greco, Carlo Talarico, Roberta Tutino, Nicola Falco, Paolina Venturelli, Gianfranco Cocorullo, Renato Pietroletti, Vinicio Rizza, Giovanni Milito, Michela Campanelli, Giorgio Lisi, Salvatore Brachitta, Venera Cavallaro, Giuseppe Pecorella, Bruno Turri, Diego Sasia, Maria Carmela Giuffrida, Marco Milone, Giovanni Palma, Vincenzo Bianco, Elisabetta Moggia, Giuseppina Talamo, Angelo Oggianu, Michela Pili, Alessio Palumbo, Marco Fazio, Domenico Aiello, Francesco Bianco, Andrea Bondurri, Gaetano Gallo, Marco Torre, Stefano Macini, Giovanni Milito, Roberto Perinotti, Renato Pietroletti, Alberto Serventi, Marina Fiorino

**Affiliations:** 1 Department of Medical and Surgical Sciences, University of Catanzaro, Catanzaro, Italy; 2 Department of Colorectal Surgery, S. Rita Clinic, Vercelli, Italy; 3 Department of General Surgery, University of Insubria, Varese, Italy; 4 Proctology and Pelvic Floor Clinical Centre, Cisanello University Hospital, Pisa, Italy; 5 Department of Advanced Biomedical Sciences, Federico II University, Naples, Italy; 6 Department of Health Sciences, University of Catanzaro, Catanzaro, Italy

**Keywords:** Haemorrhoidal disease, Post-operative pain, Open excisional haemorrhoidectomy, Mesoglycan, Thrombosis, Mucocutaneous bridges

## Abstract

**Background:**

Excisional haemorrhoidectomy is the gold standard technique in patients with III and IV degree haemorrhoidal disease (HD). However, it is associated with a significant rate of post-operative pain. The aim of our study was to evaluate the efficacy of mesoglycan in the post-operative period of patients who underwent open excisional diathermy haemorrhoidectomy (OEH).

**Methods:**

This was a retrospective multicentre observational study. Three hundred ninety-eight patients from sixteen colorectal referral centres who underwent OEH for III and IV HD were enrolled. All patients were followed-up on the first post-operative day (T1) and after 1 week (T2), 3 weeks (T3) and 6 weeks (T4). BMI, habits, SF-12 questionnaire, VAS at rest (VASs), after defecation (VASd), and after anorectal digital examination (VASe), bleeding and thrombosis, time to surgical wound healing and autonomy were evaluated.

**Results:**

In the mesoglycan group, post-operative thrombosis was significantly reduced at T2 (p < 0.05) and T3 (p < 0.005), and all patients experienced less post-operative pain at each time point (p < 0.001 except for VASe T4 p = 0.003). There were no significant differences between the two groups regarding the time to surgical wound healing or post-operative bleeding. There was an early recovery of autonomy in the mesoglycan group in all three follow-up periods (T2 p = 0.016; T3 p = 0.002; T4 p = 0.007).

**Conclusions:**

The use of mesoglycan led to a significant reduction in post-operative thrombosis and pain with consequent early resumption of autonomy.

*Trial registration* NCT04481698—Mesoglycan for Pain Control After Open Excisional HAEMOrrhoidectomy (MeHAEMO) https://clinicaltrials.gov/ct2/show/NCT04481698?term=Mesoglycan+for+Pain+Control+After+Open+Excisional+HAEMOrrhoidectomy+%28MeHAEMO%29&draw=2&rank=1

## Background

Haemorrhoidal disease (HD) is the most common proctological disease, with a prevalence that can reach up to 39% of the population [1]. Although I and II degree HD can be treated successfully with medical therapy [2] or office-based procedures [3], excisional haemorrhoidectomy remains the gold standard technique in patients with III and IV degree HD [2], obtaining a much lower rate of recurrence than non-excisional methods, such as Doppler-guided haemorrhoidal artery ligation [4] or stapled haemorrhoidopexy [5]. However, both open and closed haemorrhoidectomies are associated with a significant rate of post-operative pain [6], which may be due to the incorporation of sensitive anal mucosa and fibres of the internal sphincters during the ligation of the vascular pedicle, post-operative scars, hygiene/social habits, hard stool, or oedema of the necessary mucocutaneous bridge [7–9].

In a single-blind randomised trial comparing open excisional diathermy haemorrhoidectomy with pedicle ligation or pedicle coagulation, Bessa et al. [10] demonstrated a statistically significant reduction in post-operative pain during the first 6 post-operative days as well as a reduction in the amount of analgesics required in the group of patients undergoing pedicle coagulation.

Although radiofrequency haemorrhoidectomy is a good and safe painless alternative that does not require ligation of the vascular pedicle [11], it was recently considered an independent risk factor for delayed bleeding [12]. Furthermore, The Working Group of PROSPECT (PROcedure-SPECific post-operative pain managemenT) recommended open haemorrhoidectomy with electrocoagulation of the pedicle as the procedure of choice, especially in terms of post-operative pain [13].

Regarding the oedema/thrombosis of the mucocutaneous bridges, we strongly believe that it is the main cause of post-operative pain, and we have shown that the use of mesoglycan, a polysaccharide complex with antithrombotic and profibrinolytic properties, can reduce the rate of post-operative thrombosis and consequently post-operative pain 7–10 days after the procedures [9], improving patient quality of life and speeding up the recovery of daily activities.

Furthermore, its usefulness is also evident in the treatment of the acute phase of external haemorrhoidal thrombosis [14].

The aim of our study was to evaluate the efficacy of mesoglycan in the post-operative period of patients who underwent open excisional diathermy haemorrhoidectomy, confirming the previously obtained results [9].

## Methods

### Study design

This was a retrospective multicentre study and is reported according to the Strengthening the Reporting of Observational Studies in Epidemiology (STROBE) statement for cohort studies [15].

Data were collected and stored in an online database by the Coordinator Centre for the following: name of the study site/surgeon; BMI, habits (coffee, smoking, polypharmacy, type of diet) SF-12 questionnaire (administered before and 90 days after surgery); VAS at rest (VASs), after defecation (VASd), and after anorectal digital examination (VASe); bleeding and thrombosis; evaluation of surgical scars (granulation, time to healing); possible autonomy and time of return to work.

All patients were followed-up on the first post-operative day (T1) and three times after discharge: T2 (1 week), T3 (3 weeks), and T4 (6 weeks).

Between September and December 2017, 206 patients with III and IV degree HD, according to Goligher classification [16], from sixteen colorectal referral centres belonging to SICCR (*Società Italiana di Chirurgia Colorettale*), who satisfied inclusion and exclusion criteria (Table 1), underwent OEH.

**Table 1:** Exclusion criteria

Age < 18
Past or present history of: Coagulopathy Cardiac diseases Anticoagulant therapies Colorectal or anal neoplasms Inflammatory bowel disease Pelvic radiotherapy Anal surgery Allergy to mesoglycan
Inability to return for post-operative control visits

Due to the observational nature of this research, no formal sample size determination was performed. The minimum number of patients belonging to Mesoglycan Group (MG) was chosen based on our previous study, i.e., 10 patients [9]. A maximum of 2 investigators from each centre were included as collaborators. We have given high-volume centres the opportunity to participate with double teams.

The procedures were performed as previously described [17] with the patient in the lithotomy position and under spinal anaesthesia with removal of the three classical piles. Discharge was planned the day after surgery.

All patients received the standard post-operative therapy (a recommended oral dose of ketorolac tromethamine of 10 mg every 4–6 h, not exceeding 40 mg per day and not exceeding 5 post-operative days according to the indications for short-term management of moderate/severe acute post-operative pain and stool softeners) plus mesoglycan (Prisma^®^ 30 mg 2 vials i.m./day for the first 5 post-operative days and then Prisma^®^ 50 mg 1 oral tablet twice/day for an additional 30 days; Mediolanum Farmaceutici, Milan, Italy).

The results obtained were compared with a homogeneous sample of 192 patients who underwent OEH in the same centres between April and July 2017 and who had received standard post-operative therapy without mesoglycan.

In each referral centre, the procedures were carried out by an experienced surgeon who had performed more than 200 haemorrhoidectomies.

A clinical external examination was performed the first post-operative day, and an anorectal digital evaluation with proctoscopy was performed at T2, T3 and T4.

During each follow-up visit, post-operative pain was evaluated at rest, after defecation and after anorectal digital examination using a visual analogue scale (VAS) (minimum score = 0; maximum score = 10).

Quality of life was evaluated pre- and post-operatively 90 days after the procedure using the SF-12 questionnaire [18, 19].

Polypharmacy was defined as 5 or more medications daily.

Thrombosis was defined as one or more swollen painful piles at the site of the mucocutaneous bridge and was assessed at T2, T3 and T4.

Surgical wound healing (granulation) was evaluated at T2, T3 and T4 using the following 3 items: infected, granulating, healed.

The severity of bleeding was assessed by the number of bleeding episodes.

Bleeding was assessed using a dichotomous parameter (yes or not) and defined as persistent in cases of more than 3 episodes after day 2 following EH.

Autonomy was evaluated at T2, T3 and T4 using the following 4 items: complete inactivity, total autonomy at home, ability to drive, return to normal activities (autonomy at home, driving, working).

Bowel movements were evaluated, according to the proper guidelines, at T2, T3 and T4, and patients were classified in three categories: regular, constipation [20] or diarrhoea [21].

### Statistical analysis

Patient characteristics were analysed using Fisher’s exact test for categorical variables and either Mann–Whitney and Kruskal–Wallis tests (for independent measures) or Wilcoxon and Friedman tests (for repeated measures) for continuous variables. Descriptive results for continuous variables are expressed as the median [interquartile range (IQR)].

Contingency tables were created matching treatments and thrombosis and bleeding at each study time point, calculating the chi-square and risk ratio. SF-12 Physical component score (PSC) and Mental component score (MSC) distributions were tested for normality (Kolmogorov–Smirnov test) and then compared by Student’s t test for paired samples and represented by box plots showing median, interquartile interval, outliers and extreme values. Score deltas were compared with t tests for unpaired samples. All tests were carried out with the help of SPSS 21.0, version for Windows. A p value less than 0.05 was considered significant for all tests.

## Results

From September to December 2017 and from April to July 2017, three hundred and ninety-eight patients from 16 tertiary referral centres with III-IV degree HD underwent OEH, receiving standard post-operative therapy with (MG) or without mesoglycan (CG) (206 vs 192 pts, respectively).

No statistically significant differences were noted for age, sex, habits or grade of disease (Table 2).

**Table 2:** Patient characteristics

	Mesoglycan group (N = 206)	Control group (N = 192)
Mean age (years)	53.93 ± 15.185 (19–93)	54.27 ± 14.912 (23–91)
Sex (male)	118 (57%)	114 (59%)
Haemorrhoidal disease degree (N; %)		
III	76 (37%)	67 (35%)
IV	130 (63%)	125 (65%)
Coffee (more than 2/day)	152 (74%)	142 (74%)
Smoking (N; %)	80 (39%)	75 (39%)
Polypharmacy (N; %)	34 (16%)	28 (15%)
Diet (vegetarian)	18 (9%)	15 (8%)

No intraoperative complications or drug-related side effects occurred. All patients were discharged the day after the procedures.

Tables 3 and 4 show the incidence of post-operative bleeding and thrombosis in the two groups.

**Table 3:** Incidence of post-operative bleeding

Follow-up	CG	MG	p value
T2 (N; %)	16/192 (8.3)	14/206 (6.7)	0.562
T3 (N; %)	3/192 (1.5)	5/206 (2.4)	0.774
T4 (N; %)	0/192 (0)	0/206 (0)	–

**Table 4:** Incidence of post-operative thrombosis

Follow-up	CG	MG	p value
T2 (N; %)	24/192 (12.5)	13/206 (6.3)	< 0.05
T3 (N; %)	20/192 (10.4)	7/206 (3.3)	0.005
T4 (N; %)	4/192 (2)	2/206 (1)	0.363

**Fig. 1:**
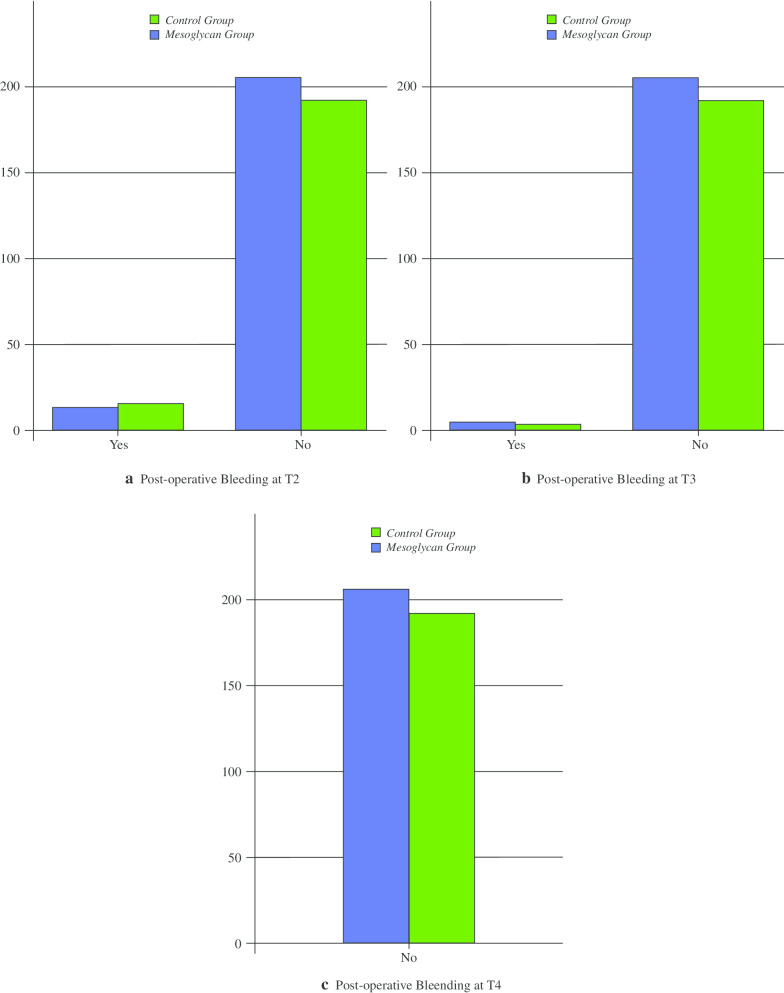
Post-operative bleeding

Post-operative bleeding was reported in 14 and 16 patients at T2 and in 3 and 5 patients at T3 in the MG and CG, respectively (Table 3; Fig. 1a, b). There were no statistically significant differences in this parameter. Furthermore, no delayed post-operative bleeding occurred at T4 in either group (Table 3; Fig. 1c). Seven MG patients and five CG patients, who experienced persistent bleeding within the first 7 post-operative days, required re-admission and re-operation, while all other cases were treated conservatively.

**Fig. 2:**
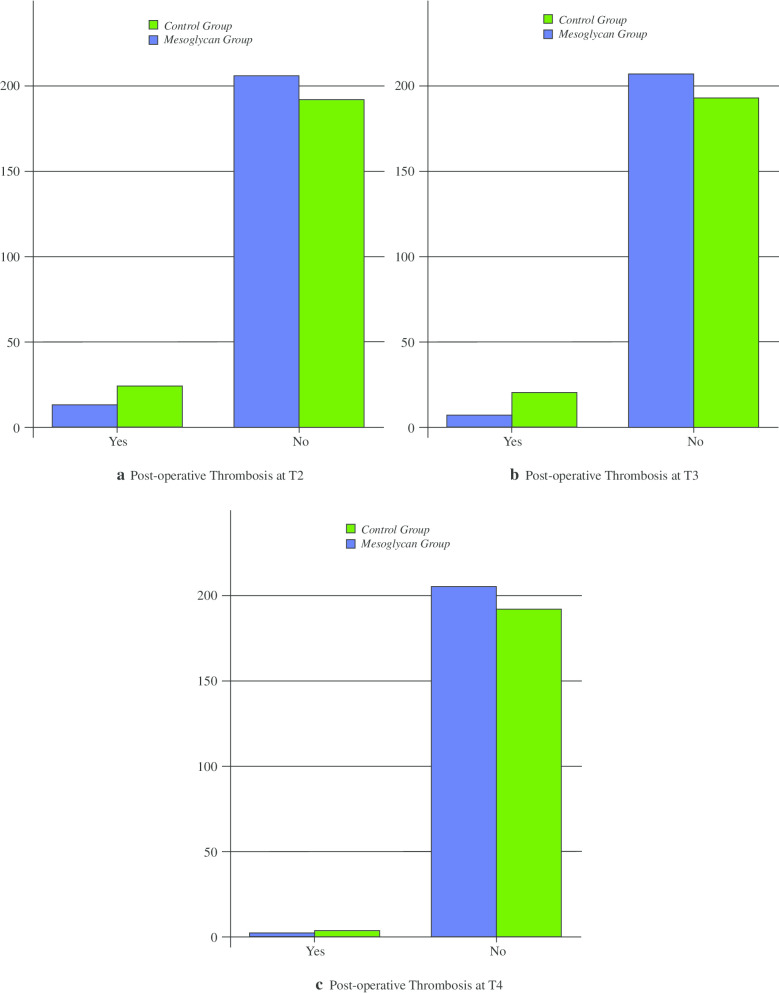
Post-operative thrombosis

A significant reduction in post-operative thrombosis was observed in MG at T2 and T3 compared with CG (p < 0.05) (Table 4; Fig. 2a, b). At T4, there were no differences between the two groups (Fig. 2c).

At each time point, there was a statistically significant reduction in VASs, VASe and VASd (p < 0.0001) for the mesoglycan-treated group with a consequent rapid recovery of the normal activities (Table 5). Only in VASe at T4 was the difference between the two groups lower (Table 6). Considering that the majority of the patients had no bowel movement at 1, VASd was not evaluated at this time. Moreover, in order to avoid any bias, VASs and VASe at T1 were not evaluated outside the aim of this study, as mesoglycan was first administered on the morning of discharge.

**Table 5:** Post-operative activities

Follow-up	Inactivity		Autonomy at home		Driving		Return to work		p value
	CG	MG	CG	MG	CG	MG	CG	MG	
T2 (N; %)	3 (1.6)	1 (0.5)	128 (66.6)	117 (57)	43 (22.3)	48 (23.3)	13 (6.8)	36 (17.4)	0.016
T3 (N; %)	0 (0)	0 (0)	45 (23.4)	52 (25.2)	55 (28.6)	29 (14)	91 (47.3)	125 (60.7)	0.002
T4 (N; %)	0 (0)	0 (0)	44 (23)	41 (20)	27 (13.1)	11 (5.3)	120 (62.5)	149 (72.3)	0.007

**Table 6:** Post-operative pain assessment

Follow-up	VAS	CG	MG	p value
T2	VASs	4.8 ± 2.61 (0–10)	3.41 ± 1.92 (0–10)	< 0.0001
	VASd	5.98 ± 2.37 (0–10)	4.98 ± 2.1 (0–10)	< 0.0001
	VASe	5.96 ± 2.9 (0–10)	4.71 ± 2.56 (0–10)	< 0.0001
T3	VASs	3.37 ± 2.6 (0–10)	1.98 ± 1.71 (0–6)	< 0.0001
	VASd	4.61 ± 2.56 (0–10)	3.4 ± 2 (0–8)	< 0.0001
	VASe	4.56 ± 2.86 (0–10)	3.5 ± 2.5 (0–9)	< 0.0001
T4	VASs	1.83 ± 1.95 (0–10)	1.1 ± 1.3 (0–5)	< 0.0001
	VASd	3 ± 2.42 (0–10)	2.0 ± 2 (0–7)	< 0.0001
	VASe	2.5 ± 2.1 (0–6)	1.9 ± 1.6 (0–6)	0.003

*VASs* post-operative pain at rest, *VASd* post-operative pain after defecation, *VASe* post-operative pain after anorectal digital examination

**Fig. 3:**
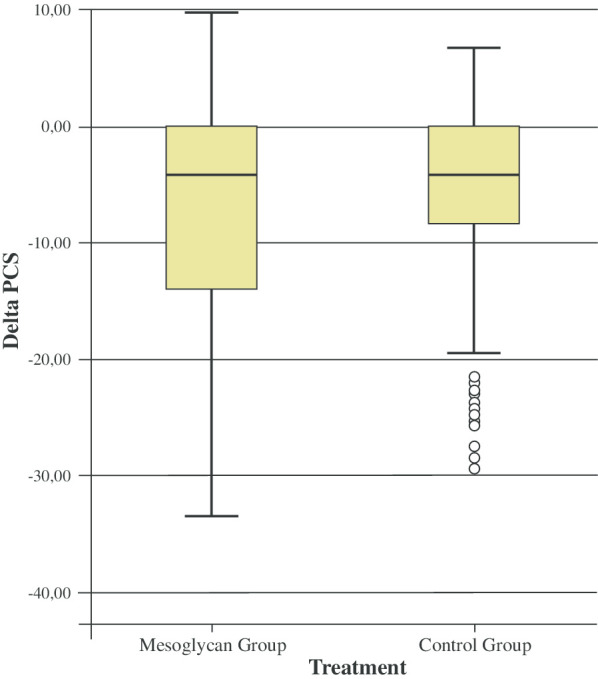
Boxplot representing the physical score (PCS) distribution of the SF-12 questionnaire

**Fig. 4:**
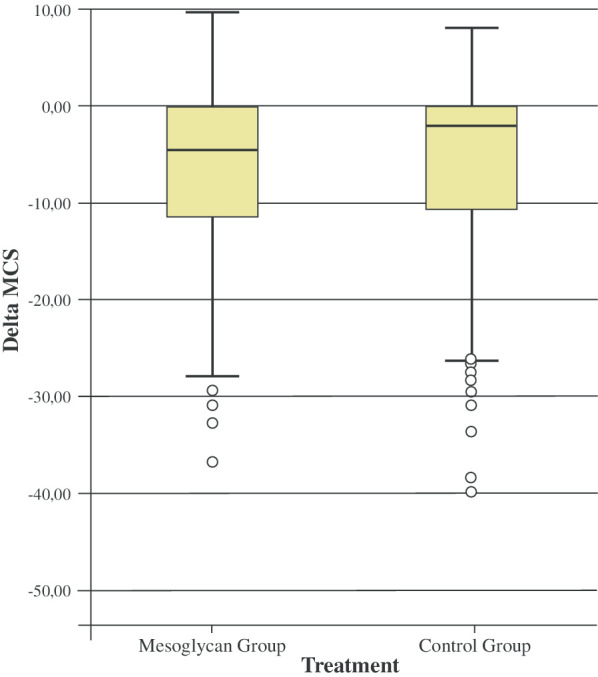
Boxplot representing the mental score (MCS) distribution of the SF-12 questionnaire

Both the physical component summary score (PCS) and mental component summary score (MCS) improved in the post-operative period in the two groups (Table 7; Figs. 3, 4).

**Table 7:** Pre- and post-operative quality of life

SF-12	Pre-operative	Post-operative	Mean difference between pre- and post-operative periods	p value
PCS-MG	44 ± 8.9	51.8 ± 5.2	7.8 ± 8.9	< 0.0001
PCS-CG	45.6 ± 8.2	51.6 ± 5.4	5.9 ± 7.5	< 0.0001
MCS-MG	48.6 ± 12	55.5 ± 7.3	6.9 ± 7.5	< 0.0001
MCS-CG	48.4 ± 12.9	54.9 ± 8.7	6.5 ± 9.5	< 0.0001

*PCS* physical component summary score, *MCS* mental component summary score

Interestingly, there was no difference between the group in the PCS (p = 0.615), whereas the MCS improvement was statistically significant in the MG (p < 0.05).

Regarding bowel movements, the trends in the two groups were different (Table 8).

**Table 8:** Bowel movements in the groups during the three follow-up periods

Follow-up	Regular		Diarrhoea		Constipation		p value
	CG	MG	CG	MG	CG	MG	
T2 (N; %)	112 (58.3)	145 (70.4)	19 (9.9)	14 (6.8)	61 (31.8)	47 (22.8)	0.055
T3 (N; %)	136 (70.8)	180 (87.4)	13 (6.8)	7 (3.4)	43 (22.4)	19 (9.2)	< 0.0001
T4 (N; %)	151 (78.6)	193 (93.7)	1 (0.5)	5 (2.4)	40 (20.8)	8 (3.9)	< 0.0001

In fact, apart from T2 (p = 0.055) in the other two follow-up periods, the differences were statistically significant (p < 0.0001).

**Fig. 5:**
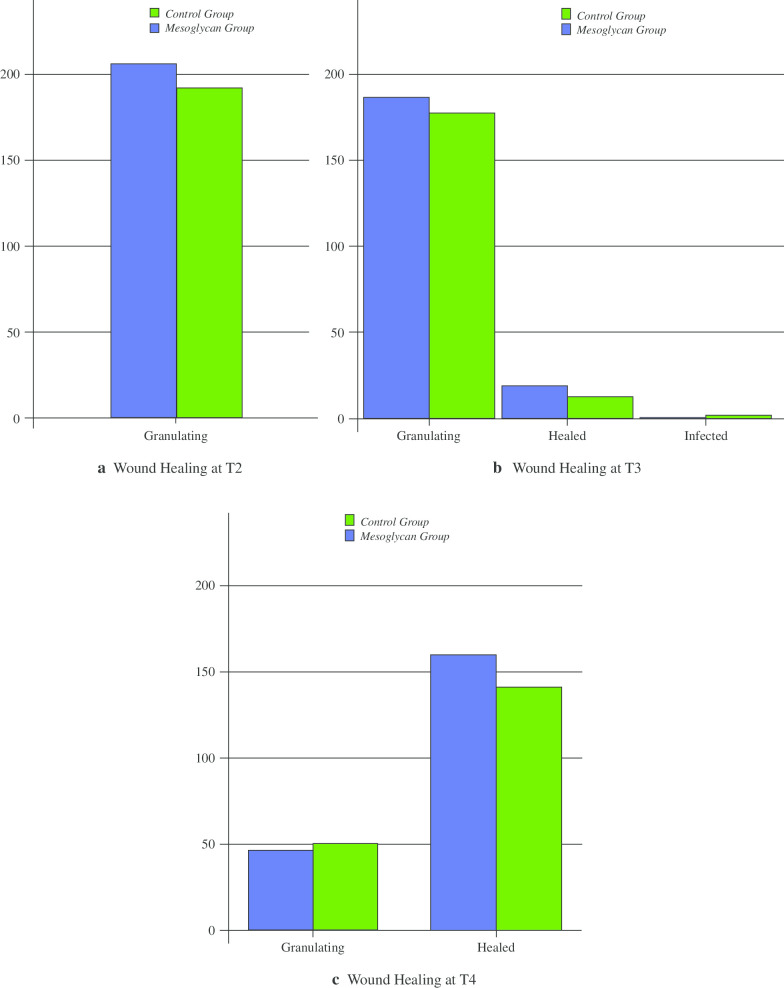
Wound healing

There were no significant differences between the two groups in the time to surgical wound healing (Table 9; Fig. 5a–c).

**Table 9:** Wound healing

Follow-up	Infected		Granulating		Healed		p value
	CG	MG	CG	MG	CG	MG	
T2 (N; %)	0 (0)	0 (0)	192 (100)	206 (100)	0 (0)	0 (0)	^a^
T3 (N; %)	2 (1)	1 (0.5)	177 (94.3)	186 (90.3)	13 (6.8)	19 (9.2)	0.551
T4 (N; %)	0 (0)	0 (0)	51 (26.6)	47 (20.8)	141 (73.4)	159 (77.1)	0.386

^a^Statistical comparison not available because of empty cases

## Discussion

Excisional haemorrhoidectomy, the so-called “Milligan-Morgan technique”, remains the most common procedure for III- and IV-degree HD [22].

Post-operative pain is one of the main topics of discussion that conditions the patient's choice not to undergo surgery. For this reason, in recent years, several techniques based on the principle of dearterialization and mucopexy have been proposed to overcome this problem. However, the recurrence rate continues to be high for these new techniques [2].

This is the second report regarding the role of mesoglycan in the post-operative period of HD. Our results confirm the antithrombotic and consequently pain-relieving action of mesoglycan.

In fact, in our first report, post-operative pain reduction was statistically significant at T2, with the vast majority of patients who had a faster return to work at T4 (93.9%—MG vs 70.5%—CG) [9].

As expected, there were no statistically significant differences in bleeding, as it was not dependent on the action of the mesoglycan, which does not alter the parameters of the coagulation but has only an antifibrinolytic effect [23], as well as on the time to surgical wound healing.

In fact, the main effect of mesoglycan is related to its antithrombotic activities on mucocutaneous bridges with post-operative pain reduction at T2, T3 and T4 and a consequent faster autonomy.

There was no difference in the thrombosis rate at T4 for the natural evolution of the post-operative period.

There was a statistically significant difference between the pre- and post-operative periods in both components of quality of life and in both groups. This result is in line with the heavy burden caused on all patients by HD from both a physical and psychological point of view.

The MCS component improved the most in the MG group (p < 0.05). In our opinion, this was probably closely related to the reduction in post-operative pain.

Almost 31%, 22% and 21% of the patients in the CG presented constipation (Table 8). Except at T2, these values were statistically significant (p < 0.001) when compared to MG, probably due to the greater post-operative pain, which constituted a limiting factor during defecation.

All procedures were performed by experienced colorectal surgeons with a standardised technique. In fact, individual surgeons have been considered independent risk factors for post-operative outcomes [24, 25]. Furthermore, there has been considerable standardisation in the evaluation of parameters in the pre- and post-operative periods. The latter, along with the high number of patients and the multicentric design, are the main strengths of our study.

However, this study has some limitations. The different number of patients between the two groups and the non-randomisation design represent the main weaknesses. Furthermore, some centres participated in the study with a double team due to the greater volume of patients enrolled.

## Conclusions

The antithrombotic properties of mesoglycan have led to a reduction in post-operative pain and an early resumption of autonomy, probably due to the reduction in thrombosis of the mucocutaneous bridges. These results have to be confirmed by a future randomised controlled trial.

## Data Availability

The datasets generated during and/or analysed during the current study are available from the corresponding Author on reasonable request.

## Abbreviations

HD: Haemorrhoidal disease
OEH: Open excisional diathermy haemorrhoidectomy
MG: Mesoglycan Group
CG: Control Group
VASs: Visual analogue scale at rest
VASd: VAS after defecation
VASe: VAS after anorectal digital examination
PCS: Physical component summary score
MCS: Mental component summary score
