# Correction to: Mesoglycan for pain control after open excisional HAEMOrrhoidectomy (MeHAEMO): an observational multicentre study on behalf of the Italian Society of Colorectal Surgery (SICCR)

**DOI:** 10.1186/s12893-020-00940-3

**Published:** 2020-11-11

**Authors:** G. Gallo, S. Di Saverio, G. Clerico, A . Sturiale, M. Manigrasso, A. Realis Luc, M. Trompetto, G. Sammarco, the MeHAEMO Working Group, Francesco Ferrari, Antonio Carpino, Giuseppe Sena, Giuseppina Vescio, Emanuela Stratta, Alberto Realis Luc, Giuseppe Clerico, Mario Trompetto, Paolo Tonello, Silvia Cornaglia, Vincenzo Greco, Carlo Talarico, Roberta Tutino, Nicola Falco, Paolina Venturelli, Gianfranco Cocorullo, Renato Pietroletti, Vinicio Rizza, Giovanni Milito, Michela Campanelli, Giorgio Lisi, Salvatore Brachitta, Venera Cavallaro, Giuseppe Pecorella, Bruno Turri, Diego Sasia, Maria Carmela Giuffrida, Marco Milone, Giovanni De Palma, Vincenzo Bianco, Elisabetta Moggia, Giuseppina Talamo, Angelo Oggianu, Michela Pili, Alessio Palumbo, Marco Fazio, Domenico Aiello, Francesco Bianco, Andrea Bondurri, Gaetano Gallo, Marco La Torre, Stefano Mancini, Giovanni Milito, Roberto Perinotti, Renato Pietroletti, Alberto Serventi, Marina Fiorino

**Affiliations:** 1 Department of Medical and Surgical Sciences, University of Catanzaro, Catanzaro, Italy; 2 Department of Colorectal Surgery, S. Rita Clinic, Vercelli, Italy; 3 Department of General Surgery, University of Insubria, Varese, Italy; 4 Proctology and Pelvic Floor Clinical Centre, Cisanello University Hospital, Pisa, Italy; 5 Department of Advanced Biomedical Sciences, Federico II University, Naples, Italy; 6 Department of Health Sciences, University of Catanzaro, Catanzaro, Italy

## Correction to: BMC Surg (2020) 20:251 10.1186/s12893-020-00914-5

Following publication of the original article, we were notified that three of the names of the MeHAEMO working group were incorrectly reported. The correct names are: Marco La Torre, Stefano Mancini and Giovanni De Palma.

The original article has been corrected.

